# Management of cardiovascular risk factors with pioglitazone combination therapies in type 2 diabetes: an observational cohort study

**DOI:** 10.1186/1475-2840-10-18

**Published:** 2011-02-11

**Authors:** Ángel Rodríguez, Jesús Reviriego, Vasilios Karamanos, Francisco J del Cañizo, Nikolaos Vlachogiannis, Vangelis Drossinos

**Affiliations:** 1 Clinical Research Department, Lilly, S.A., Avda. de la Industria, 30, 28108 Alcobendas, Madrid, Spain; 2 Diabetes Center, 2nd Department of Internal Medicine and Research Laboratory, National University of Athens, Hippokration General Hospital, Vas Sofias 114, 11527 Ambelokipoi, Athens, Greece; 3 Department of Endocrinology and Nutrition, University Hospital Infanta Leonor, School of Medicine, Universidad Complutense, Gran Vía del Este, 80, 28031 Madrid, Spain; 4 Health Center of Lavrion, General Hospital of Athens "G. Gennimatas", Mesogeion 154, 11527 Athens, Greece; 5 Department of Medical Research, Pharmaserve-Lilly S.A.C.I., National Route Athens-Lamia, 14564 Kifissia, Greece

## Abstract

**Background:**

Type 2 diabetes (T2D) is strongly associated with cardiovascular risk and requires medications that improve glycemic control and other cardiovascular risk factors. The authors aimed to assess the relative effectiveness of pioglitazone (Pio), metformin (Met) and any sulfonylurea (SU) combinations in non-insulin-treated T2D patients who were failing previous hypoglycemic therapy.

**Methods:**

Over a 1-year period, two multicenter, open-labeled, controlled, 1-year, prospective, observational studies evaluated patients with T2D (n = 4585) from routine clinical practice in Spain and Greece with the same protocol. Patients were eligible if they had been prescribed Pio + SU, Pio + Met or SU + Met serving as a control cohort, once they had failed with previous therapy. Anthropometric measurements, lipid and glycemic profiles, blood pressure, and the proportions of patients at microvascular and macrovascular risk were assessed.

**Results:**

All study treatment combinations rendered progressive 6-month and 12-month lipid, glycemic, and blood pressure improvements. Pio combinations, especially Pio + Met, were associated with increases in HDL-cholesterol and decreases in triglycerides and in the atherogenic index of plasma. The proportion of patients at high risk decreased after 12 months in all study cohorts. Minor weight changes (gain or loss) and no treatment-related fractures occurred during the study. The safety profile was good and proved similar among treatments, except for more hypoglycemic episodes in patients receiving SU and for the occurrence of edema in patients using Pio combinations. Serious cardiovascular events were rarely reported.

**Conclusions:**

In patients with T2D failing prior hypoglycemic therapies, Pio combinations with SU or Met (especially Pio + Met) improved blood lipid and glycemic profiles, decreasing the proportion of patients with a high microvascular or macrovascular risk. The combination of Pio with SU or Met may therefore be recommended for T2D second-line therapy in the routine clinical practice, particularly in patients with dyslipidemia.

## Background

Type 2 diabetes mellitus (T2D) is a progressive and heterogeneous disease associated with macrovascular and microvascular complications that increase morbidity and mortality [1]. Cardiovascular disease (CVD) risk is higher in patients with T2D than in the matched nondiabetic population [2]. About half of deaths in patients with T2D are caused by CVD, predominantly due to ischemic heart disease [3]. Although controlling hyperglycemia is the primary goal for T2D treatments, appropriate T2D management requires addressing multiple comorbidities. Effective interventions to revert dyslipidemia, hypercoagulation and hypertension, in addition to hyperglycemia, have been shown to reduce diabetic complications and mortality [4].

Pioglitazone (Pio) belongs to the group of thiazolidinediones (TZDs), agents that diminish insulin resistance primarily by selective binding to peroxisome-proliferator-activated receptor-γ (PPAR-γ). TZDs have been recommended as a second-line therapy in patients with T2D that fail to achieve therapeutic goals while on monotherapy, particularly when they show multiple metabolic derangements on top of hyperglycemia [4,5]. The PPAR-γ receptor regulates expression of genes involved in glucose, fatty acid, and cholesterol metabolism, and plays a critical role in the vasculature [6]. It has been suggested that Pio reduces hyperglycemia by enhancing insulin sensitivity through PPAR-γ binding, but it also influences lipid metabolism and represses the endothelial inflammatory gene expression involved in atherosclerosis development by binding PPAR-α receptors [7,8]. Nonetheless, there is a debate surrounding the long-term risk/benefit ratio of TZDs, because TZDs' pleiotropic effects, derived from the many actions of the genes activated by PPAR-γ agonism, may entail side effects which would diminish or even counteract their positive effects, resulting in a net increase in the risk of macrovascular complications [9].

The high prevalence of diabetic dyslipidemia makes many patients with T2D candidates for receiving second-line combined treatment with Pio. However, few randomized studies have compared the commonly prescribed oral hypoglycemic combination of a sulfonylurea (SU) plus metformin (Met) (SU + Met) with the Pio combinations Pio + SU [10] or Pio + Met [11,12]. There has only been one randomized study directly comparing the aforementioned 3 combinations [13], and one observational study comparing Pio + Met versus SU + Met [14], and neither evaluated lipid profile. A subanalysis of the Prospective Pioglitazone Clinical Trial in Macrovascular Events (PROactive) in patients with T2D and established CVD evaluated the lipid profile of the 3 combinations, among others, but no comparisons were performed among them; rather, Pio + SU and Pio + Met were compared with the respective SU and Met monotherapies plus placebo, and SU + Met + Pio was compared with SU + Met + placebo [15].

The present report corresponds to the data pooled from two 1-year prospective, observational, controlled cohort studies. Investigators evaluated the effectiveness of Pio combinations, with SU or Met, compared to the control combination of SU plus Met, in improving metabolic derangements (including overweight/obesity, dyslipidemia, hyperglycemia and arterial hypertension) in a large sample of patients with T2D in routine clinical practice in Spain and Greece.

## Methods

### Study design and patients

Two multicenter, controlled, 1-year, prospective, observational cohort studies were performed in Spain [16] and Greece [17] with the same protocol. To obtain a representative sample, physicians specializing in T2D management from all regions of these countries were invited to participate. Eligible patients had to be 18 years of age or older, of either gender, and had to present a documented diagnosis of T2D that had been inadequately controlled with their current hypoglycemic therapy. These patients were newly prescribed with a specific combined therapy (Pio + SU, Pio + Met, or SU + Met). The Pio doses required to participate had to be 15 or 30 mg/day. Patients with conditions or medications contraindicated with study drugs, or patients requiring insulin therapy, were excluded.

Patients were included in the study if they were prescribed 1 of the study therapies during routine clinical visits. Cohort size was balanced by including blocks of 9 patients (3 per cohort), following the decision to prescribe any of the 3 drug combinations. Investigators were advised not to commence a new block unless the previous one was completed.

The study protocol complied with the Declaration of Helsinki and regulations for postauthorization observational studies in Spain and Greece, and was approved by accredited Ethics Committees from both countries. All patients signed and dated written Informed Consent Forms, and data was collected in Case Report Forms where anonymity was maintained.

### Assessments and procedures

Procedure at baseline entailed the collection of sociodemographic, anthropometric, clinical, and laboratory data. This included information about the age of T2D diagnosis and T2D duration, measurement of fasting plasma glucose (FPG) and glycosylated hemoglobin (HbA1c), serum high-density lipoprotein cholesterol (HDL-C), total cholesterol (total-C), low-density lipoprotein cholesterol (LDL-C) and triglycerides, systolic (SBP) and diastolic (DBP) blood pressures, cardiovascular history (arterial hypertension, microvascular and macrovascular disorders, peripheral arterial occlusive disease), and measurements of hepatic (alanine transaminase, ALT) and renal (serum creatinine) functions.

Effectiveness was measured by changes in several biochemical tests for blood obtained at 3 time points: during the 30 days prior to starting the combined study therapies (baseline assessment), and at the 6-month (±1) and 12-month (±1) follow-up visits. Because only a few studies in T2D patients have focused on patients' lipid profiles, and given the authors' interest in macrovascular risk, the HDL-C was considered the primary endpoint.

All the laboratory measurements were assessed after an overnight fast. HDL-C, total-C, and triglycerides were assayed by enzymatic methods, and LDL-C was estimated using the Friedewald equation. FPG was measured by means of a glucose-oxidase-based method. HbA1c was determined by a high-performance liquid chromatographic method. Local clinical laboratories assayed all tests using their working standards. Raw results were standardized against the reference ranges from the laboratory that analyzed more samples. SBP and DBP were measured by using a standard brachial cuff technique after having the patient rest for 10 minutes in a seated position.

In addition, some estimations regarding cardiovascular risk were performed for the 3 moments in which the patients were assessed: 1) quotient total-C/HDL-C and atherogenic index of plasma (AIP), calculated as log (triglycerides/HDL-C) [18]; 2) categorization of patients into cardiovascular risk ranges estimated by the European Diabetes Policy Group (EDPG), based on glycemic and lipid profiles [19]; and 3) achievement of targets for diabetic patients related to lipid profile according to both EDPG [19] and International Diabetes Federation (IDF) recommendations [4].

Weight, measured to the nearest 0.1 kg, alanine transaminase (ALT), and creatinine were also monitored throughout the study. Safety was also supervised by recording hypoglycemic episodes and adverse events (AEs). AEs were coded using the Medical Dictionary for Regulatory Activities (MedDRA), version 3.3.

### Statistical methods

Sample size was calculated at 2190 patients for each country to provide a 90% power to detect a difference of at least 0.05 mmol/l between 2 means of HDL-C using a two-group t-test with a 0.05 two-sided significance level. This sample size considered 3 cohorts of patients and assumed a 40% withdrawal rate and a common standard deviation of 0.24 mmol/l.

Analyses of effectiveness included data from patients who started study treatments and had at least 1 postbaseline assessment. Missing values were calculated using the last observation carried forward (LOCF) method. As a sensitivity analysis, efficacy was also evaluated in the actual (observed) 12-month values.

Baseline variables were summarized by descriptive statistics, as were changes of the study variables (standardized biochemical tests, blood pressure, weight, and AIP) from baseline to the 6-month and 12-month visits.

Vascular and cardiovascular risks across cohorts were evaluated by examining the proportion of patients in each EDPG risk range (observed 12-month data) for glycemic and blood lipid tests [19]. Compliance with low-risk EDPG [19] and IDF [4] lipid profile goals was expressed as a percentage difference of cohort means above or below target levels. Frequencies of hypoglycemic episodes and AEs were compared by chi-square or Fisher exact tests.

The EDPG cardiovascular risk ranges were also used to estimate the likelihood of being at low risk versus higher risk levels by logistic regression analyses done separately for each relevant glycemic and blood lipid component (HbA1c, FPG, HDL-C, total-C, LDL-C and triglycerides). These models adjusted for the study treatment, gender, age, baseline weight, duration of diabetes, usage of lipid-lowering drugs (for blood lipid profile), and baseline levels of each modeled component.

## Results

### Patients and treatments

**Figure 1:**
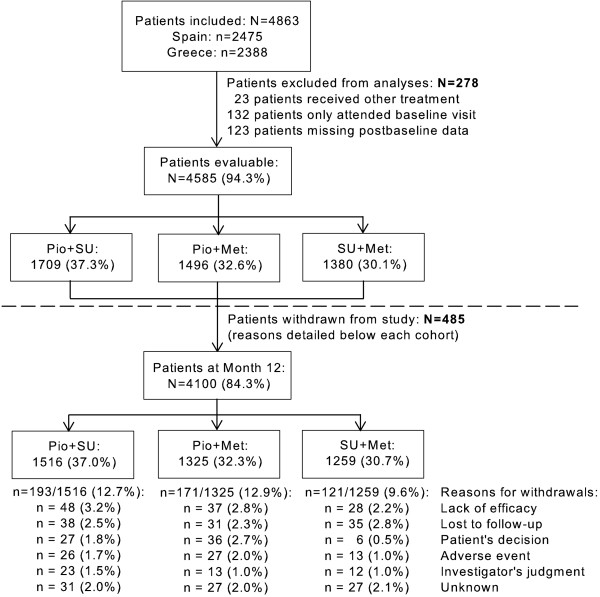
**Distribution of patients throughout the study**.

Between May 2002 and November 2005 4585 patients were evaluated. In total, 485 left the study prematurely. The proportion of patients withdrawing prematurely was slightly greater in the Pio cohorts. Withdrawal causes were similar among cohorts, except when it came to consent withdrawals, which were more frequent with Pio. Figure 1 shows patients' distribution throughout the study.

Previous hypoglycemic agents were mainly SU (54%) or Met (43%). Most patients maintained their prior hypoglycemic drug with the new added agent. Pio was given at 30 mg daily doses except for 18.5% of patients receiving 15 mg/day, with few changes by Month 12. The most prescribed SUs were similarly distributed among cohorts; glimepiride, gliclazide, and glibenclamide were the most common. The same median dose for Met and the most prescribed SUs were maintained in all cohorts (Met: 1700 mg/day; glimepiride: 4 mg/day; gliclazide: 160 mg/day; and glibenclamide: 15 mg/day).

Some patients concomitantly received lipid-lowering agents (27% Pio + SU, 28% Pio + Met, 29% SU + Met) and/or antihypertensive therapy (44% Pio + SU, 40% Pio + Met, 45% SU + Met) throughout the whole study.

### Baseline assessments

Patients' baseline characteristics are shown in Table 1. Mean total-C and LDL-C were above the low EDPG risk range and FPG and HbA1c above the nondiabetic range. Physicians' prescription criteria were guided by the clinical status of individual patients, resulting in differences between cohorts on some baseline variables. Patients prescribed with Pio + Met had an overall worse clinical status in terms of cardiovascular risk factors, including a greater body mass index (BMI), than patients of other cohorts. Patients treated with Pio + SU had the lowest baseline weight, BMI, and proportion of obesity, but the highest FPG and HbA1c. Patients receiving SU + Met had the lowest baseline FPG and HbA1c.

**Table 1:** Patients' characteristics at baseline (N = 4585)

	Pio + SU n = 1709	Pio + Met n = 1496	SU + Met n = 1380
***Sociodemographics***			

Age, years	62.5 ± 9.8 [3]	58.7 ± 10.5 [4]	62.2 ± 9.8 [5]
Gender, females	869 (50.8) [0]	795 (53.1) [0]	733 (53.1) [0]
Smoker, yes	333 (19.6) [7]	341 (22.9) [4]	273 (19.9) [8]

***Anthropometrics***			

Height, cm	164 ± 9 [0]	165 ± 9 [1]	164 ± 9 [2]
Weight, kg	77 ± 13 [0]	85 ± 15 [1]	79 ± 14 [2]
BMI,^† ^kg/m^2^	28.8 ± 4.5 [0]	31.2 ± 5.2 [1]	29.6 ± 4.6 [2]
Obese patients (BMI ≥30 kg/m^2^)	532 (31.1) [0]	777 (52.0) [1]	537 (39.0) [2]

***Glycemic profile***			

Age T2D diag.,* years	53.9 ± 10.4 [8]	51.7 ± 10.4 [15]	53.8 ± 10.2 [10]
T2D duration, years	8.6 ± 7.0 [10]	7.0 ± 6.3 [19]	8.4 ± 6.9 [11]
FPG, mmol/l	10.8 ± 2.5 [77]	10.4 ± 2.6 [55]	10.2 ± 2.5 [56]
HbA1c, %	8.4 ± 1.7 [59]	8.2 ± 1.6 [50]	8.0 ± 1.6 [43]

***Lipid profile***			

HDL-C, mmol/l	1.18 ± 0.28 [114]	1.17 ± 0.30 [82]	1.19 ± 0.28 [95]
LDL-C, mmol/l	3.99 ± 0.69 [207]	3.94 ± 0.71 [218]	3.96 ± 0.69 [192]
Total-C, mmol/l	5.58 ± 1.12 [58]	5.59 ± 1.14 [56]	5.47 ± 1.14 [59]
Triglycerides, mmol/l	2.05 ± 0.83 [56]	2.15 ± 0.86 [63]	2.00 ± 0.81 [41]
AIP	0.59 ± 0.22 [160]	0.61 ± 0.21 [135]	0.57 ± 0.21 [134]

***Cardiovascular status***			

SBP, mm Hg	142 ± 19 [8]	142 ± 18 [5]	143 ± 18 [3]
DBP, mm Hg	82 ± 10 [8]	84 ± 10 [5]	83 ± 10 [3]
Arterial hypertension	999 (58.5) [0]	867 (58.0) [0]	865 (62.7) [0]
Microvascular history	320 (19.1) [34]	265 (18.0) [27]	301 (22.3) [33]
Macrovascular history	328 (19.2) [0]	256 (17.1) [0]	303 (22.0) [0]
*Angina pectoris*	153 (9.0) [0]	128 (8.6) [0]	146 (10.6) [0]
*Myocardial infarction*	110 (6.6) [34]	58 (3.6) [33]	86 (6.4) [32]
*Heart failure*	49 (2.9) [0]	42 (2.8) [0]	68 (4.9) [1]
*Ictus*	63 (3.8) [31]	52 (3.6) [34]	64 (4.7) [30]
*Perip. arterial occlusive disease*	125 (7.5) [39]	119 (8.1) [33]	130 (9.6) [31]

***Hepatic and renal function***			

ALT, IU/l	28.6 ± 11.5 74	29.4 ± 12.0 84	29.2 ± 11.8 71
Creatinine, μmol/l	90 ± 25 [44]	89 ± 23 [45]	90 ± 24 [55]

Values are either the numbers of patients with their percentages within parenthesis or means ± standard deviations. Values within brackets indicate the numbers of patients with missing data for that variable.

* Age at diagnosis of T2D. † BMI classification according to the Spanish Society for the Study of Obesity (SEEDO) [55].

Values are either number of patients with their percentages within parentheses or mean ± standard deviation. Values within brackets indicate the number of patients with missing data for that variable.

### Evolution of blood lipids

**Figure 2:**
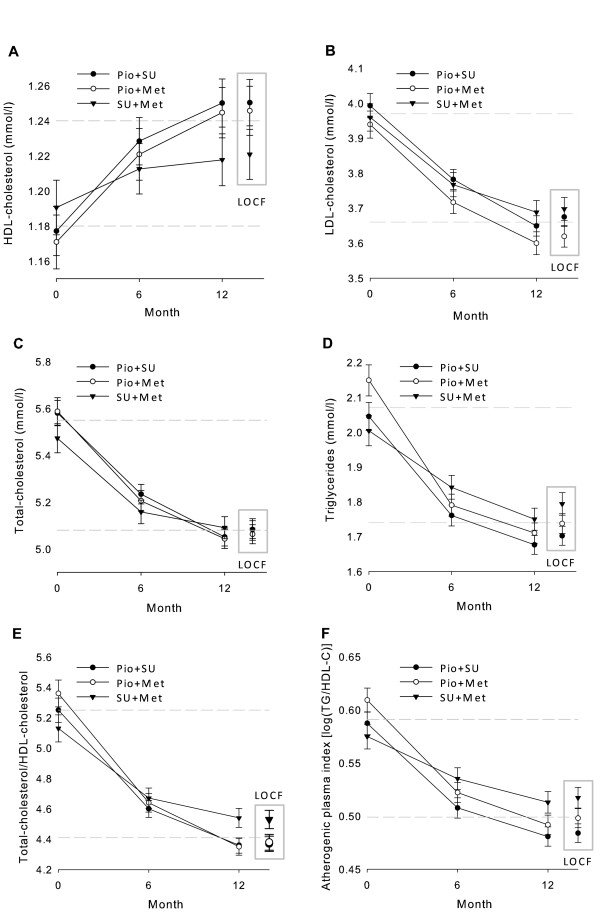
**Evolution of HDL-cholesterol (A), LDL-cholesterol (B), total-cholesterol (C), triglycerides (D), total-cholesterol/HDL-cholesterol (E), and atherogenic index of plasma (F), from baseline to Month 12**. Data points are mean values and error bars represent 95% CIs. The 2 dashed horizontal lines mark the range between mean levels of each variable for the total population at baseline and at Month 12 (LOCF data).

Baseline to Month 12 changes in HDL-C (Figure 2A and Table 2) showed a progressive increase that was about two times greater with Pio combinations than with SU + Met.

**Table 2:** Changes in lipid measurements and atherogenic index of plasma from baseline to Month 12

	Pio + SU n = 1709	Pio + Met n = 1496	SU + Met n = 1380
**HDL-C**	[108]	[96]	[85]

Change in means, %	6	6	3
Change in medians, %	7	9	2
Mean change, mmol/l	0.07	0.08	0.03

**LDL-C**	[128]	[125]	[127]

Change in means, %	-8	-8	-7
Change in medians, %	-5	-6	-5
Mean change, mmol/l	-0.32	-0.32	-0.26

**Total-C**	[70]	[70]	[72]

Change in means, %	-9	-9	-7
Change in medians, %	-7	-8	-5
Mean change, mmol/l	-0.51	-0.54	-0.39

**Triglycerides**	[53]	[54]	[39]

Change in means, %	-17	-19	-10
Change in medians, %	-15	-18	-9
Mean change, mmol/l	-0.35	-0.41	-0.21

**AIP**	[135]	[124]	[105]

Change in means, %	-18	-18	-10
Change in medians, %	-18	-18	-9
Mean change	-0.11	-0.12	-0.06

Values within brackets indicate the number of patients with missing data for that variable. The numbers represent the percentage changes in the mean and median values, and the absolute mean change, from baseline to Month 12 (LOCF data).

The other lipid profile components decreased progressively (Figures 2B to 2F and Table 2). Differences among cohorts on LDL-C and total-C were less important. Triglycerides also decreased more with Pio combinations, especially Pio + Met. In all cohorts, lipid profile changes were more pronounced among patients who received concomitant lipid-lowering medications than among those who did not (data not shown).

### Evolution of glycemic variables

**Figure 3:**
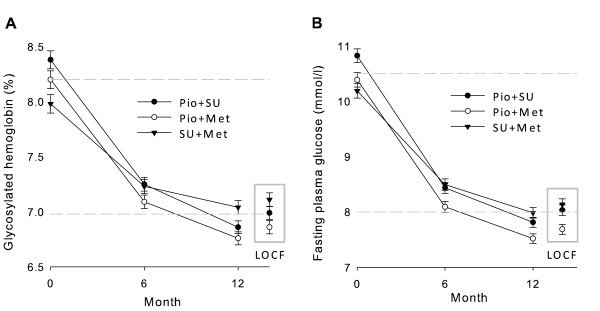
**Evolution of glycosylated hemoglobin (A) and fasting plasma glucose (B) from baseline to Month 12**. Data points are mean values and error bars represent 95% CIs. The 2 dashed horizontal lines mark the range between baseline and Month 12 (LOCF data) mean levels of each variable for the total population.

The 12-month reductions in HbA1c (mean [SD] reductions: -1.3 [1.4] % Pio + SU, -1.3 [1.3] % Pio + Met, -0.9 [1.3] % SU + Met; Figure 3A), and FPG (-2.7 [0.1] mmol/l in Pio cohorts vs -2.1 [0.1] mmol/l with SU + Met; Figure 3B) were both greater with Pio combinations than with SU + Met.

### Evolution of blood pressure

**Figure 4:**
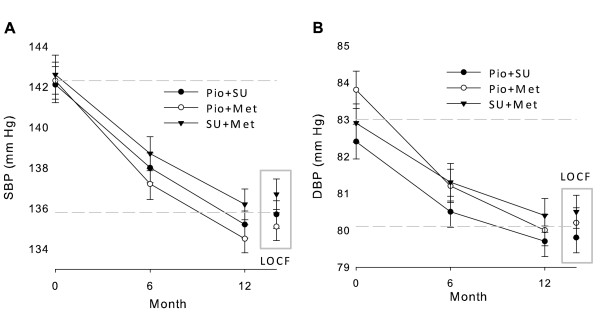
**Evolution of systolic (SBP, panel A) and diastolic (DBP, panel B) blood pressures from baseline to Month 12**. Data points are mean values and error bars represent 95% CIs. The two dashed horizontal lines mark the range between mean levels of each variable for the total population at baseline and at Month 12 (LOCF data).

There were slight reductions of both systolic (SBP) and diastolic blood pressure (DBP) measurements, in all cohorts (Figure 4).

### Evolution of anthropometric variables

Mean weight and BMI values in Pio + Met were the highest at baseline (Table 1) and throughout the whole study (data not shown). There were only minor changes after 12 months resulting in a mean weight gain with Pio + SU (+ 0.8 kg), and a weight decrease with Pio + Met (-0.7 kg) and SU + Met (-0.9 kg).

### Changes in cardiovascular risk assessments

Progressive 12-month reductions of AIP were greater with Pio treatments (Figure 2F).

**Figure 5:**
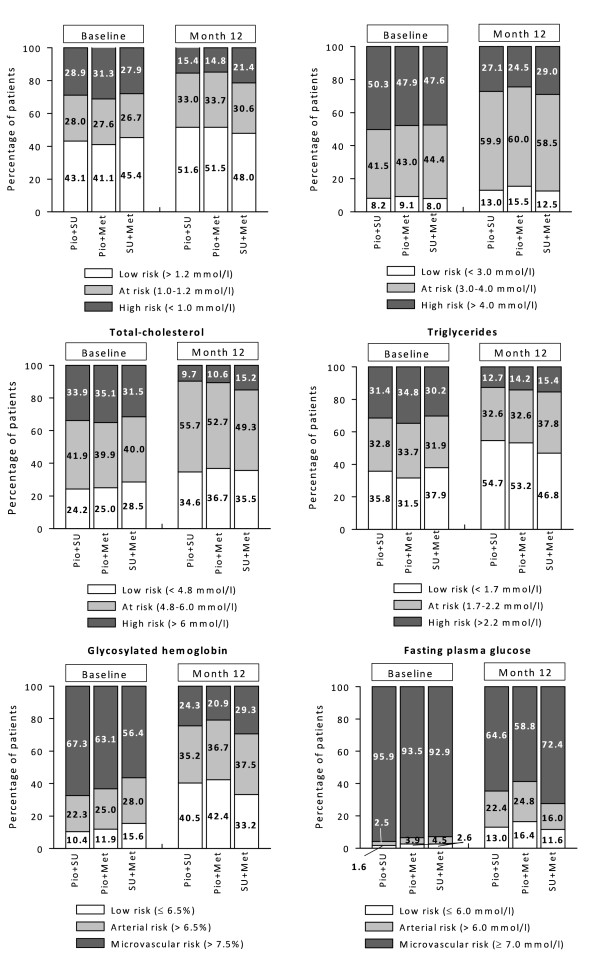
**Proportion of patients distributed according to the risk ranges for cardiovascular events and vascular complications estimated by the EDPG, related to lipid profile (A-D) and glycemic status (E-F)**.

Considering EDPG risk ranges, the proportions of patients at low risk increased in all cohorts after 12 months, in particular regarding total-C, triglycerides, and glycosylated hemoglobin (Figure 5). In general, the increases of patients at low risk were more noticeable with Pio combinations.

Logistic regression analyses revealed a consistently lower likelihood of being in high risk EDPG categories for Pio cohorts for HDL-C (odds ratios versus the SU + Met cohort: 0.671, 95% CI: 0.545 - 0.827 Pio + SU; 0.672, 0.539 - 0.837 Pio + Met), triglycerides (0.597, 0.500 - 0.713 Pio + SU; 0.559, 0.463 - 0.674 Pio + Met), HbA1c (0.528, 0.440 - 0.633 Pio + SU; 0.595, 0.494 - 0.717 Pio + Met), and FPG (0.771, 0.607 - 0.981 Pio + SU; 0.654, 0.514 - 0.830 Pio + Met). For total-C, lower likelihood was only significantly associated with receiving Pio + Met (0.799, 0.654 - 0.974) and not significant with Pio + SU (0.898, 0.742 - 1.086). Results related to LDL-C were not significantly different among the study cohorts.

**Figure 6:**
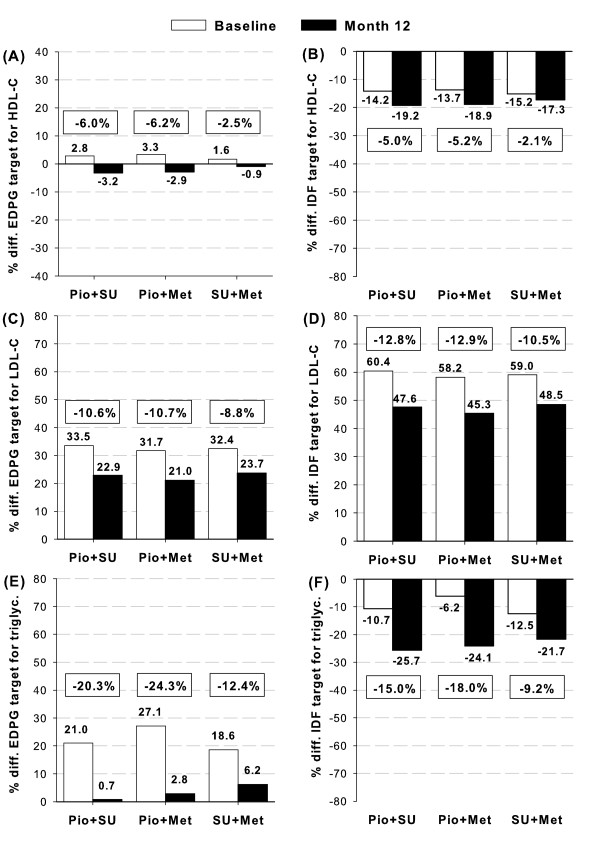
**Percentage deviations of mean values of lipid profile from the recommended targets for diabetic patients according to the criteria for low-risk EDPG range (panels A, C, E) **[19]** and IDF lipid targets (panels B, D, F) **[4]. Panels A-B: HDL-C, C-D: LDL-C, and E-F: triglycerides. Level zero marks the cutpoint for each component, values over zero indicate percentage differences over unfulfilled target levels, and values below zero are the percentage differences over fulfilled target levels. HDL-C values have been reversed accordingly. Framed numbers indicate percentage changes from baseline to Month 12 within each cohort.

Compliance with EDPG and IDF lipid profile goals improved from baseline (Figure 6). Mean HDL-C and triglycerides reached the low risk range (as per EDPG) or surpassed target levels (as per the IDF) after 12 months of treatment. Mean LDL-C approached but did not reach target levels.

### Tolerability and safety

Table 3 summarizes the main safety data. Among patients receiving SUs, there was a higher incidence of patients recording at least one hypoglycemic episode. Few hypoglycemic episodes were severe, these occurred at similar frequencies among cohorts.

**Table 3:** Summary of the main safety data during the study

	Pio + SU n = 1709	Pio + Met n = 1496	SU + Met n = 1380
**Hypoglycemic episodes**			

At least one	175 (10.3) [2]	91 (6.1) [6]	134 (9.7) [1]
Severe	5 (0.3) [36]	4 (0.3) [36]	5 (0.4) [26]

**Adverse events**	[0]	[0]	[0]

At least one adverse event	113 (6.6)	98 (6.6)	73 (5.3)
Related to study treatments*	70 (4.1)	60 (4.0)	28 (2.0)
*Gastrointestinal disorders*	13 (0.8)	15 (1.0)	15 (1.1)
*General disorders*	28 (1.6)	25 (1.7)	3 (0.2)
Edema	26 (1.5)	22 (1.5)	2 (0.1)
At least one serious adverse event	13 (0.8)	10 (0.7)	12 (0.9)
Description of all serious AEs (by body system):			
*Cardiac disorders*	4 (0.2)	2 (0.1)	2 (0.1)
*Gastrointestinal disorders*	-	-	1 (0.1)
*General disorders*	-	-	1 (0.1)
*Infections and infestations*	-	2 (0.1)	-
*Injury, poisoning, and procedural complications*	1 (0.1)	-	-
*Metabolism and nutrition disorders*	1 (0.1)	-	-
*Neoplasm*	2 (0.1)	1 (0.1)	2 (0.1)
*Nervous system disorders*	3 (0.2)	2 (0.1)	2 (0.1)
*Renal and urinary disorders*	-	-	1 (0.1)
*Reproductive system and breast disorders*	-	1 (0.1)	-
*Respiratory, thoracic, and mediastinal disorders*	1 (0.1)	-	-
*Skin and subcutaneous tissue disorders*	1 (0.1)	1 (0.1)	-
*Surgical and medical procedures*	1 (0.1)	1 (0.1)	1 (0.1)
*Vascular disorders*	-	-	2 (0.1)

* Listed if >1% in at least one cohort. Values within brackets indicate the number of patients with missing data for that variable. Data represent the numbers of patients, with percentages within parentheses.

Few patients (284/4585, 6.2%) had at least 1 AE during the study, evenly distributed among cohorts. The incidence of related AEs was low, although higher in Pio cohorts, with significant differences (p = 0.003) among cohorts. The most common related AEs were gastrointestinal disorders, similarly distributed among cohorts; and general disorders, with higher proportion in the Pio cohorts, primarily edema. There were two incidental hip fractures in the Pio + SU group that the investigators considered as unrelated to study treatments.

At least 1 serious AE was reported in 35 patients (0.8%), without differences among cohorts. Six patients (0.1%), 2 per cohort, had an AE resulting in death, but none were related to the study treatments in the physicians' criteria.

### Sensitivity analyses

Twelve-month data showed greater benefits with Pio combinations, especially with Pio + Met, than LOCF data (Figures 2 to 4). The progressive 12-month improvements were predictably blunted whenever some Month 6 data were included in the LOCF analyses.

## Discussion

In this prospective, observational, controlled cohort study in patients with T2D who had failed to achieve therapeutic goals in prior therapy, clinical and biochemical variables associated with cardiovascular risk progressively improved after 12-month combined treatments. These improvements were greater in patients treated with Pio combinations, particularly Pio + Met, than with the control combination SU + Met. In line with the results from prior randomized trials with Pio [10-12,15,20-23], such benefits were especially noticeable for the rise in HDL-C and the decline in triglycerides.

Decreased HDL-C, increased LDL-C and triglycerides, hyperglycemia, hypertension, and smoking have classically been reported as independent risk factors for CVD in patients with T2D [24]. The major cardiovascular and diabetes organizations have established target levels to be achieved by diabetic patients for LDL-C, HDL-C, and triglycerides [4,19,25,26].

Although lowering LDL-C to target has been the main goal to reduce the risk of CVD in the general population [27], LDL-C particle size, in addition to total concentration, has been associated with the pathogenesis of atherosclerosis and cardiovascular risk [28]. Pio treatments positively affect the T2D atherogenic profile by significantly increasing the size of LDL and HDL particles [29,30]. Thus an additional benefit of Pio could be expected from its effects over LDL subfractions, which are not visible by total LDL-C measurements. In contrast, a considerable residual cardiovascular risk still remains, largely attributed to low HDL-C and high triglycerides in diabetic patients [27,31], and concerns have been expressed regarding the ability of statins to correct the atherogenic dyslipidemia of patients with T2D, which is characterized by low HDL-C levels, elevated triglycerides, and predominance of small, dense LDL particles [32]. The AIP is a surrogate marker of atherosclerosis inversely correlated with LDL particle size [18,33]. In the present study, as in prior research [34-36], reductions of AIP were observed in the cohorts treated with Pio combinations. Moreover, because AIP is inversely correlated with insulin sensitivity [36], this data together suggests that the insulin-sensitizing role of Pio would provide an antiatherogenic effect by targeting atherogenic dyslipidemia. Recent reports have further reinforced the role of Pio to reduce the risk of CVD through its effects on other surrogate markers, such as the carotid intima-media thickness [37] and the atheroma plaque size [38]. Therefore, current evidence supports that improvements in dyslipidemia (increasing HDL-C and reducing triglycerides and AIP), as observed in the present study, might render significant antiatherogenic effects, potentially diminishing the risk for macrovascular complications.

Although the evidence just mentioned suggests that the pleiotropic effects of Pio provide a benefit over a range of metabolic disturbances in patients with T2D, the actual potential of TZDs to reduce the risk of macrovascular complications beyond improvement of glycemic and lipid profiles remains yet to be proven. This would require the investigation of clinical endpoints in long-term prospective studies that are not yet available [39]. While the results of the PROactive trial pointed in that direction in patients with established CVD [40-42], there is no evidence published in this regard among the general population of patients with T2D [43-46]. The results from the present study are of particular interest for the Pio + Met combination as they suggest that additive or, speculatively, synergistic nonhypoglycemic effects may occur to improve metabolic disturbances related to cardiovascular risk in patients with T2D. Metformin has been shown to have relevant positive effects on hard clinical endpoints [47,48] and, despite the reports of an enhanced risk of heart failure associated to TZD use [49], Pio has been associated with reducing the risk of nonfatal myocardial infarction, death, and stroke [40,50]. Additionally, several meta-analyses failed to show an association between Pio and increased cardiovascular risk [50,51], and the fluid retention related to TZD treatment has not been associated with an increased risk of cardiovascular death similar to that associated with congestive heart failure secondary to left ventricular dysfunction [46]. Furthermore, echocardiography evaluations during Pio treatment have reported small and inconsistent effects [52,53], supporting the notion that TZD do not have a direct impact on heart muscle [54]. Notwithstanding, caution should be exerted in susceptible patients, and the assessment of specific diagnostic markers readily available in the routine practice, such as the assay of natriuretic peptide levels [52], may be of help before starting TZD therapy in patients without manifest cardiac disease. In this study, the Pio + Met combination was started mostly by patients who were previously on Met monotherapy, and these were generally overweight or obese. Switching from SU monotherapy to the Pio + Met combination might represent an advantage to some patients who are routinely switched to SU + Met when they start combined therapy after failing to achieve therapeutic goals while on SU monotherapy. Whether or not this strategy would decrease the rate of major cardiovascular events should be tested in a long-term, randomized clinical trial.

Although a limitation of this report is that it is based on pooled data from 2 separate studies, both were performed almost simultaneously with the same protocol in two Mediterranean countries with similar lifestyle habits. Thus, their results may be regarded as if they came from a single, multinational study. Nonrandomized studies, like this, provide a lower level of evidence than randomized ones. But it in turn they do offer the advantage of reflecting the actual effectiveness of treatments, given that nonrandomization bias prescription occurs in clinical practice. Other strengths include the naturalistic dosing and the use of a spectrum of SUs in contrast to previous clinical trials of TZDs. In general, patients in the Pio + Met cohort presented a poorer clinical condition, so it is possible that biases related to the regression-to-the-mean phenomenon occur. It is also posible that other unobserved variables may have accounted for the differences among groups. As this is not a clinical outcome study, it does not contribute answers to the issue of cardiovascular prevention in patients with T2D treated with TZDs. Also, among the cardiovascular risk factors that the TZDs have the potential to improve (other than dyslipoproteinemias), such as endothelial dysfunction, inflammation, fibrinolysis, or arterial hypertension, only the latter was examined in this study. Finally, although the occurrence of fractures in this study was incidental, there is an association established between treatment with Pio and an increased risk of bone fractures in the long term. Whether new fractures would have been reported in these patients should they have been followed for a longer time period is unknown.

## Conclusions

Beyond a beneficial effect in glycemic control in patients with T2D, Pio combinations evaluated in this study also improved various metabolic variables associated with cardiovascular risk. Importantly, Pio therapies were associated with potentially greater antiatherogenic protection than SU + Met. While demonstration of actual macrovascular risk reduction requires a long-term assessment, the findings from this study in real clinical settings support the use of these Pio combinations for T2D second-line therapy, particularly in patients with dyslipidemia.

## Abbreviations

AE: adverse event; AIP: atherogenic index of plasma; ALT: alanine transaminase; ANOVA: one-way analysis of variance; BMI: body mass index; CVD: cardiovascular disease; DBP: diastolic blood pressure; EDPG: European Diabetes Policy Group; CI: confidence interval; FPG: fasting plasma glucose; HbA1c: glycosylated hemoglobin; HDL-C: high-density lipoprotein cholesterol; IDF: International Diabetes Federation; LDL-C: low-density lipoprotein cholesterol; LOCF: last observation carried forward; MedDRA: Medical Dictionary for Regulatory Activities; Met: metformin; OR: odds ratio; Pio: pioglitazone; PPAR: peroxisome proliferator-activated receptor; PROactive: Prospective Pioglitazone Clinical Trial in Macrovascular Events; SBP: systolic blood pressure; SD: standard deviation; SEEDO: Spanish Society for the Study of Obesity; SU: sulfonylurea; T2D: type 2 diabetes; total-C: total cholesterol; TZD: thiazolidinediones.

## Competing interests

AR, and JR are full-time employees of Lilly, SA, Spain, and VD is a full-time employee of Pharmaserve-Lilly SACI, Greece. These companies are corporate affiliates of Eli Lilly and Company.

FC has participated and received honoraria as clinical investigator from Lilly, Merck-Sharp-Dohme, Sanofi-Aventis, Novo-Nordisk, and Boehringer-Ingelheim, and has received honoraria for his participation in lectures and events sponsored by Lilly, Novo-Nordisk, Sanofi-Aventis, Merck-Sharp-Dohme, Novartis, Almirall, Glaxo-Smith-Kline, Bayer, Abbott, Bristol-Myers-Squibb, Astra Zeneca, Menarini, Roche, Daiichi-Sankyo, Schering-Plough, and Servier.

VK has received research support and honoraria as speaker from Astra Zeneca, Eli Lilly and Novo.

NV has received grants as clinical investigator from Lilly and Sanofi.

## Authors' contributions

AR participated in protocol design, supervised the preparation of the manuscript and the statistical analyses, and approved the final version.

JR and VD participated in protocol design, performed a critical review of the manuscript for important intellectual content, and approved the final version.

FC, VK, and NV participated in the acquisition of clinical data, performed a critical review of the manuscript for important intellectual content, and approved the final version.

## Supplementary Material

Additional file 1 **Appendix**. List of investigators participating in the ECLA (Evaluation of the Clinical effects on the Lipid profile of oral Antidiabetics) study. Click here for file
