# Supplementary material for: Management of cardiovascular risk factors with pioglitazone combination therapies in type 2 diabetes: an observational cohort study
Source: Cardiovasc Diabetol. 2011 Feb 11;10:18. doi: 10.1186/1475-2840-10-18 (PMC3042924; doi:10.1186/1475-2840-10-18)
Supplement: Additional file 1 — Appendix. List of investigators participating in the ECLA (Evaluation of the Clinical effects on the Lipid profile of oral Antidiabetics) study. [file 1475-2840-10-18-S1.DOC]

# Management of cardiovascular risk factors with pioglitazone combination therapies in type 2 diabetes: An observational cohort study

**Ángel Rodríguez, Jesús Reviriego, Vasilios Karamanos, Francisco José del Cañizo, Nikolaos Vlachogiannis, Vangelis Drossinos, on behalf of the ECLA study group**

**APPENDIX**

Investigators participating in the ECLA (**E**valuation of the **C**linical effects over the **L**ipid profile of oral **A**ntidiabetics) study are listed by country in alphabetical order:

**List of Investigators from Spain:**

**Alberto García Valdés -** Hospital Militar del Aire, MADRID; **Alberto Gilsanz Peral** - Hospital La Fe, C. VALENCIA; **Alfonso Arranz Martín -** Hospital Ntra. Sra. de Sonsoles, C. LEÓN; **Alfonso Calañas Continente -** Hospital General Reina Sofía, ANDALUCÍA; **Alfonso Gentil Baldrich -** Hospital Univ. Virgen Macarena, ANDALUCÍA; **Alfonso González-Cruz Cervellera -** Hospital General Univ. Valencia, C. VALENCIA; **Alfonso Pumar López -** Centro Periférico Especialidades Virgen de los Reyes, ANDALUCÍA; **Alfredo Luis Michan Doña -** Hospital Jerez de la Frontera, ANDALUCÍA; **Alicia Picón Martínez -** Ambulatorio Bola Azul, ANDALUCÍA; **Alicia Serraclara Pla -** Ambulatorio de Villaverde Cruce, MADRID; **Amparo Marco Martínez -** Hospital Virgen de la Salud, C. MANCHA; **Amparo Segura Galindo -** Ambulatorio Peña Pietra, MADRID; **Andreu Nubiola Calonge** - Hospital Espíritu Santo, CATALUÑA; **Ángel González Amieva -** Hospital Valle de los Pedroches, ANDALUCÍA; **Antonio Acosta Socorro** - Hospital Gral. de Gran Canaria Dr. Negrín, CANARIAS; **Antonio Becerra Fernández -** Centro de Especialidades San Blas, MADRID; **Antonio Campayo Ibáñez -** Hospital de Onteniente, C. VALENCIA; **Antonio Espino Montoro -** Hospital de la Merced, ANDALUCÍA; **Antonio Fernández Moyano** - Hospital San Sebastián, ANDALUCÍA; **Antonio Hernández López / Cristina Lamas Oliveira -** Complejo Hospital Gral. de Albacete, C. MANCHA; **Antonio López-Guzmán Guzmán** - Hospital Ntra. Sra. de Sonsoles, C. LEÓN; **Antonio Miguel Hernández Martínez -** Hospital Virgen de la Arrixaca, MURCIA; **Antonio Picó Alfonso -** Hospital General Alicante, C. VALENCIA; **Antonio Pozuelo González -** Hospital 12 de Octubre, MADRID; **Antonio Robles Iniesta** - Hospital General de Requena, C. VALENCIA; **Antonio Vicente Casanova -** Hospital Clínico Univ. Valencia, C. VALENCIA; **Aurelia Villar Bonet -** Hospital Univ. Clínico de Valladolid, C. LEÓN; **Azucena Rodríguez Robles -** Hospital Nuestra Sra. de Alarcos, C. MANCHA; **Basilio Moreno Esteban -** Hospital Gregorio Marañón, MADRID; **Benito Blanco Samper -** Hospital Ntra. Sra. Del Prado, C. MANCHA; **Blas Gil Extremera** - Hospital Clínico San Cecilio, ANDALUCÍA; **Braulio León Villaverde -** Hospital Virgen de las Nieves, ANDALUCÍA; **Cándido Rodríguez Vázquez** - Hospital Provincial de Badajoz, EXTREMADURA; **Carlos de Gorospe Pérez-Jáuregui** - Consulta Privada, MADRID; **Carlos Jericó Alba -** Hospital del Mar de Barcelona, CATALUÑA; **Carlos Lobón Cerviá** - Centro de Especialidades Delicias, C. LEÓN; **Carlos Sánchez Juan** - Hospital de Sagunto, C. VALENCIA; **Carmelo Gómez Aparicio -** Hospital Virgen de la Luz, C. MANCHA; **Carmen de Miguel Canalejas -** Consulta Endocrinología y Nutrición, MADRID; **Carmen Fajardo Montañana** - Hospital de la Ribera, C. VALENCIA; **Carmen Tusón Rovira -** Hospital de Cabueñes, ASTURIAS; **Ceferino Martínez Faedo** - Hospital Álvarez Buylla, ASTURIAS; **Concepción Blanco Carrera -** Hospital Príncipe de Asturias, MADRID; **Concepción Valerón Martel -** Ambulatorio Casa del Mar, CANARIAS; **Cristina Hernández Herrero -** Hospital Univ. Virgen Macarena, ANDALUCÍA; **Daniel Antonio de Luis Román** - Hospital Univ. Río Hortega, C. LEÓN; **Daniel Cepero García** - Hospital Torrecárdenas, ANDALUCÍA; **David Lorente Garces -** Consulta Privada, C. VALENCIA; **Diego Bellido Guerrero** - Hospital Naval del Ferrol, GALICIA; **Diego Godoy Rocati -** Hospital General Univ. Valencia, C. VALENCIA; **Diego Gómez Reyes -** Consulta Privada, MADRID; **Diego Miragaya García** - Hospital La Línea, ANDALUCÍA; **Eduardo Faure Nogueras -** Hospital Clínico de Zaragoza Lozano Blesa, ARAGÓN; **Eduardo Guerrero Martínez -** Hospital Río Carrión, C. LEÓN; **Enric Ballestar Mas** - Hospital de Mataró, CATALUÑA; **Enrique Castro Martínez** - Hospital Ntra. Sra. de la Montaña, EXTREMADURA; **Enrique Costilla Martín** - Hospital Gral. Univ. de Guadalajara, C. MANCHA; **Enrique González Sarmiento -** Hospital Univ. Clínico de Valladolid, C. LEÓN; **Enrique Hernández Alonso -** Hospital Naval del Mediterráneo, MURCIA; **Enrique Rodilla Sala -** Hospital de Sagunto, C. VALENCIA; **Felipe Molina Molina -** Hospital Ciudad de Jaén, ANDALUCÍA; **Fernando Ferrón Vidán -** Hospital del Conxo, GALICIA; **Fernando Losada Viñau** - Centro Periférico Especialidades Virgen de los Reyes, ANDALUCÍA; **Ferran Rius Riu -** Hospital Arnau de Vilanova, CATALUÑA; **Florentino Casal Álvarez** - Hospital de Cabueñes, ASTURIAS; **Francisco Javier Ampudia Blasco -** Hospital Clínico Univ. Valencia, C. VALENCIA; **Francisco Javier del Cañizo Gómez -** Centro de Especialidades Montes de Barbanza, MADRID; **Francisco Javier del Cazo Cativiela -** Hospital García Orcoyen, NAVARRA; **Francisco Javier Maravall Royo -** Hospital Arnau de Vilanova, CATALUÑA; **Francisco Javier Novoa Mogollón -** Hospital Insular, CANARIAS; **Francisco José Pérez Blanco -** Hospital Clínico Univ. San Cecilio, ANDALUCÍA; **Francisco José Pomares Gómez -** Hospital Clínico de San Juan, C. VALENCIA; **Gabriel Macanás Botia** - Hospital Ntra. Sra. del Rosell, MURCIA; **Ginés Gascón Ramón** - Hospital La Plana, C. VALENCIA; **Gloria Cervello Donderis** - Consulta Privada, C. VALENCIA; **Gonzalo Piédrola Maroto -** Hospital Virgen de las Nieves, ANDALUCÍA; **Ignacio García Puente** - Hospital Gral. de Gran Canaria Dr. Negrín, CANARIAS; **Ignacio Martín Suárez** - Hospital Juan Ramón Jiménez, ANDALUCÍA; **Ignacio Martínez Usó -** Centro de Especialidades de Sueca, C. VALENCIA; **Ignacio Ramos Casamayor -** Hospital Vega Baja, C. VALENCIA; **Iraida Gómez-Lobo González -** Ambulatorio de Orcasitas, MADRID; **Isaac Levy Mizrahi -** Hospital Clinic i Provincial de Barcelona, CATALUÑA; **Isidoro Carmona de Torres -** Clínica Sagrado Corazón, ANDALUCÍA; **Jacinto Fernández Pardo** - Hospital General Univ., MURCIA; **Jaime Binimelis Vidal** - Clínica Juaneda. Consulta Privada, BALEARES; **Javier Espiga Alzola** - Fundació Hospital de Mollet, PAÍS VASCO; **Javier Izaguirre Anduaga** - Hospital de la Princesa, MADRID; **Javier Peñafiel Martínez** - Hospital Torrecárdenas, ANDALUCÍA; **Javier Rivera Guzmán** - Hospital Provincial de Córdoba, ANDALUCÍA; **Javier Santamaría Sandi** - Hospital de Cruces Baracaldo, PAÍS VASCO; **Jesús Lorente Campos** - Hospital Militar San Carlos, ANDALUCÍA; **Jesús Mayos Pérez -** Fundació Sanitaria d'Igualada, CATALUÑA; **Jesús Yanini Viana** - Consulta Privada, C. VALENCIA; **Joaquín Martí Colomer** - Hospital Francesc de Borja, C. VALENCIA; **Joaquín Pechuan Asensio -** Consulta Particular, ANDALUCÍA; **Joaquín Serrano Gotarredona** - Hospital Verge dels Lliris, C. VALENCIA; **Jordi Anglada Barceló -** Hospital Mutua de Tarrasa, CATALUÑA; **Jordi Mesa Manteca** - Hospital Vall de Hebrón, CATALUÑA; **Jorge Portillo Martín** - Ambulatorio de Especialidades Jaime I, C. VALENCIA; **Jorge R. García Pérez -** Instituto Social de la Marina. Casa del Mar, ANDALUCÍA; **Jorge Reverter Calatayud -** Hospital Germans Trias i Pujol, CATALUÑA; **Jorge Sales Sanz** - Hospital Vega Baja, C. VALENCIA; **José Antonio Rubio García -** Hospital Príncipe de Asturias, MADRID; **José Aurelio Fernández Álvarez -** Hospital Costa Burela, GALICIA; **José Contreras Gilbert** - Hospital Univ. Virgen Macarena, ANDALUCÍA; **José Francisco López Martínez** - Hospital Rafael Méndez, MURCIA; **José Luis Griera Borras** - Hospital Univ. Virgen Macarena, ANDALUCÍA; **José Luis Pinzón Martín -** Hospital Univ. Virgen de la Victoria, ANDALUCÍA; **José Manuel Badenas Sierra -** Consulta Privada, MADRID; **José Manuel García-Almeida -** Hospital Clínico Málaga, ANDALUCÍA; **José Manuel Miralles García -** Hospital Univ. de Salamanca, C. LEÓN; **José Manuel Querol Ribelles** - Hospital Lluis Alcanyis, C. VALENCIA; **José Manuel Quesada Gómez** - Hospital Provincial de Córdoba, ANDALUCÍA; **José Manuel Ruiz Palomar** - Centro de Especialidades San Blas, C. VALENCIA; **José Miguel Braza Ramos -** Instituto Social de la Marina. Casa del Mar, ANDALUCÍA; **José Ramón Calabuig Alborch -** Hospital La Fe, C. VALENCIA; **José Ramón Domínguez Escribano -** Hospital Clínico de San Juan, C. VALENCIA; **José Sabán Ruiz** - Hospital Ramón y Cajal, MADRID; **José Villar Ortiz** - Hospital Virgen de Rocío de Sevilla, ANDALUCÍA; **José Zurro Hernández -** Hospital Univ. Clínico de Valladolid, C. LEÓN; **Josefina García Reinoso -** Hospital Arnau de Vilanova, C. VALENCIA; **Juan Ángel Hernández Bayo** - Hospital General de La Palma, CANARIAS; **Juan Antonio Arazola Pérez** - Consulta Privada, ANDALUCÍA; **Juan Antonio García Arnés -** Complejo Hospitalario Carlos Haya, ANDALUCÍA; **Juan Salmerón de Diego -** Hospital Gregorio Marañón, MADRID; **Julio Nájera Mortes -** Consulta Privada, C. VALENCIA; **Julio Ruiz de Gordejuela** - Hospital Bajo Deba, PAÍS VASCO; **Livio Héctor Peña Guillermo -** Centro de Especialidades Monteoliveti, C. VALENCIA; **Luis Ciprés Casasnovas -** Hospital Gral. de Teruel Obispo Polanco, ARAGÓN; **Luis Enríquez Acosta -** Hospital San Pedro de Alcántara, EXTREMADURA; **Luis Escobar Jiménez -** Consulta Privada, ANDALUCÍA; **Luis Fernando Vences Benito -** Centro Especialidades San Fermín, C. VALENCIA; **Luis Francisco Santiago Peña** - Hospital Virgen de la Concha, C. LEÓN; **Luis García Pascual -** Hospital Mutua de Tarrasa, CATALUÑA; **Luis Miguel Álvarez Aragón -** Hospital de Osuna, ANDALUCÍA; **Luis Montenegro Rodríguez** - Consulta Privada, ANDALUCÍA; **Luis Morcillo Herrera -** Hospital Univ. de Canarias (Laguna), CANARIAS; **Luis Teigell García** - Hospital Gregorio Marañón, MADRID; **Mª Ángeles Bueno Cascón** - Clínica Granada Salud. (ADESLAS), ANDALUCÍA; **Mª Ausencia Tomé Martínez de Rituerto** - Hospital del Conxo, GALICIA; **Mª Beatriz Flandez González** - Hospital de Getafe, MADRID; **Mª Concepción Terroba Larumbe** - Hospital Univ. Río Hortega, C. LEÓN; **Mª José Salas Romero** - Consulta Privada, ANDALUCÍA; **Mª Natividad Moreira Andrés -** Ambulatorio de Villaverde Cruce, MADRID; **Mª Teresa García Ingelmo** - Hospital Ciudad de Coria, EXTREMADURA; **Manuel Álvarez Fernández** - Consulta Privada, ASTURIAS; **Manuel Blanco Suárez** - Clínica Sagrado Corazón, ANDALUCÍA; **Manuel Calero Fresneda** - Hospital Univ. Puerto Real, ANDALUCÍA; **Manuel Cornejo Barrera** - Hospital Jerez de la Frontera, ANDALUCÍA; **Manuel León de Lope** - Hospital Juan Ramón Jiménez, ANDALUCÍA; **Manuel Muñoz Rodríguez** - Hospital de León, C. LEÓN; **Manuel Muñoz Torres -** Hospital Clínico San Cecilio, ANDALUCÍA; **María del Pilar Lamela Estévez** - Ambulatorio Hermanos Miralles, MADRID; **María Jesús Chinchetru Ranedo -** Hospital Txagorritxu, PAÍS VASCO; **María José Picón Cesar** - Hospital Clínico Málaga, ANDALUCÍA; **Mariano Villa Bautista** - Hospital de Móstoles, MADRID; **Marta Fernández Fernández** - Hospital del Bierzo, C. LEÓN; **Martín López de la Torre Casares** - Hospital Virgen de las Nieves, ANDALUCÍA; **Mercè Bergua Llop** - Ambulatorio Prat de la Riba, CATALUÑA; **Mercedes Pascual Díaz** - Hospital J. M. Morales Meseguer, MURCIA; **Mercedes Rodríguez del Palacio Fdez.** - Consulta Privada, CANARIAS; **Mercedes Tolosa Torrens** - Consulta Privada, C. VALENCIA; **Miguel Aguirre Sánchez-Covisa** - Hospital Nuestra Sra. de Alarcos, C. MANCHA; **Miguel Ángel Mangas Cruz -** Centro Periférico Especialidades Virgen de los Reyes, ANDALUCÍA; **Miguel Ángel Martínez Olmos** - Hospital Meixoeiro, GALICIA; **Miguel Ángel Rico Corral -** Hospital Univ. Virgen Macarena, ANDALUCÍA; **Miguel Civera Andrés -** Hospital de Xativa y Onteniente, C. VALENCIA; **Mónica Lorenzo Solar -** Hospital del Conxo, GALICIA; **Nieves Carretero Rodrigo -** Hospital de la Cruz Roja, MADRID; **Nuria Valdés Gallego** - Instituto Social de la Marina, ASTURIAS; **Orosia Bandrés Nivela** - Hospital Comarcal de Calatayud, ARAGÓN; **Pablo Fernández Catalina -** Hospital Montecelo, GALICIA; **Pedro Montoro Marín -** Centro de Especialidades "El Carmen", MURCIA; **Pedro Valdivielso Felices -** Hospital Clínico Málaga, ANDALUCÍA; **Petra de Diego Poza** - Hospital Ntra. Sra. Del Prado, C. MANCHA; **Rafael Marín Lama** - Hospital Provincial de Córdoba, ANDALUCÍA; **Ramón Albero Gamboa** - Hospital Miguel Servet, ARAGÓN; **Ramón Arteaga Fuentes** - Hospital de Galdakao, PAÍS VASCO; **Raquel Mateo Lobo** - Hospital Santa Bárbara, C. MANCHA; **Ricard Torras Rodergas -** Hospital General de Catalunya, CATALUÑA; **Ricardo Chamorro Prado -** Hospital Nuestra Sra. de Alarcos, C. MANCHA; **Rigoberto Corrales Cruz** - CAP Tarragonés, CATALUÑA; **Rosa Burgos Peláez -** Clínica Quirón, CATALUÑA; **Rosa Cámara Gómez** - Hospital La Fe, C. VALENCIA; **Rosa Villar Vicente** - Hospital Gutiérrez Ortega, C. MANCHA; **Rosario de la Pedraja Murgoitio** - Hospital de Sierrallana, CANTABRIA; **Santiago Tofé Povedano -** Hospital Son Dureta, BALEARES; **Teresa Muros de Fuentes** - Hospital Virgen de las Nieves, ANDALUCÍA; **Vicente Campos Alborg** - Hospital Univ. La Fe, C. VALENCIA; **Víctor García-Hierro González-Regueral** - Hospital Virgen del Puerto Plasencia, EXTREMADURA; **Víctor Manuel Andia Melero** - Hospital Ntra. Sra. de Sonsoles, C. LEÓN; **Virginia Muñoz Leira -** Hospital Provincial de Pontevedra, GALICIA.

**List of Investigators from Greece:**

**Adamidis Sotirios –** ATHENS; **Adamopoulos Dimitrios –** ATHENS; **Afendakis Constantinos –** ATHENS; **Aggelopoulos Vasilios –** THESSALONIKI; **Alexiadis Stavros –** SERRES; **Andronis Christos –** ATHENS; **Anthimidis George –** CHALKIDIKI; **Antonopoulos Andreas –** ELEFSINA; **Avraam George –** ATHENS; **Ballis Dimitrios –** ATHENS; **Bavea-Krubholtz Bettina –** ATHENS; **Beskou-Vlachaki Εftychia –** ATHENS; **Biblis Ιoannis –** ATHENS; **Chiotis Nikolaos –** ATHENS; **Chiotis Stavros –** TRIKALA; **Demislis Constantinos –** VOLOS; **Dimitriadis Constantinos –** KAVALA; **Dimitsikoglou Nikolaos –** SERRES; **Ekonomidou Christina –** KARDITSA; **Falireas Nikolaos –** ATHENS; **Floros George –** IOANNINA; **Gabriel Patrick –** ISTIEA; **Georgali Androula –** SYROS; **Giamalis Dimitrios –** ATHENS; **Giannakakis Ioannis –** IOANNINA; **Giannakidis Stavros –** KOZANI; **Girtzis Ioannis –** ALEKSANDROUPOLI; **Goni Maria Eleni –** LIVADIA; **Grigoriou Achilleas –** ATHENS; **Groutsis Theodoros –** ATHENS; **Harilas Efstathios –** KIMI; **Hasban Taisir –** ELEFSINA; **Hatzilia Anna –** ATHENS; **Ioannidis Ioannis –** ATHENS; **Kagas Spyridon –** ATHENS; **Kagelaris Nikolaos –** ATHENS; **Kalpakis Nikolaos –** LAGKADAS; **Karamanos Vasilios –** ATHENS ; **Karaouzas Athanasios –** LARISSA; **Karapiperis Antonios –** LARISSA; **Karassavidou Larissa –** ATHENS; **Kassos Dimitrios –** LARISSA; **Katinakis Aleksandros –** VOLOS; **Katsaros Thomas –** ATHENS; **Kirgiannis Athanasios –** ORESTIADA; **Kiriakopoulos Constantinos –** ATHENS; **Kiriazopoulos Sotirios –** KAVALA; **Kleftaki Aikaterini –** ATHENS; **Komplionas Constantinos –** LAMIA; **Kontogiannis Anastasios –** ATHENS; **Kotis Emmanouil –** THESSALONIKI; **Kotsa Calliopi –** KOMOTINI; **Koulouri-Pinotsi Andriani –** ATHENS; **Kourtoglou George –** THESSALONIKI; **Krikaki Aikaterini –** ATHENS;  **Laina Aggeliki –** IRAKLIO; **Lalos Vasilios –** LARISSA; **Lazaridis Athanasios –** THESSALONIKI; **Likoudi Akrivi –** ATHENS; **Malafis Panagiotis –** ATHENS; **Maleza Avgi –** EDESSA; **Manes Christos –** THESSALONIKI; **Manis Ioannis –** LARISSA; **Marinos Εmmanouil –** SAPES; **Milionis Αnastasios –** ATHENS; **Misichronis George –** ATHENS; **Mitakos Νikolaos –** ALIVERI; **Mitsiou Christos –** ATHENS; **Mountzouris Dimitrios –** ATHENS; **Nikiforou Stavroula –** ATHENS; **Panopoulos George –** ATHENS; **Panos Aggelos –** ATHENS; **Papadima Sofia –** PREVEZA; **Papadimitrakis Christoforos –** IRAKLIO; **Papageorgiou George –** LAKONIA; **Papaikonomou Panagiotis –** THESSALONIKI; **Papanastasiou Stefanos –** TRIKALA; **Pappas Constantinos –** ATHENS; **Patatsi Vasiliki –** ATHENS ; **Perakakis George –** ATHENS; **Petridis Sotirios –** KASTORIA; **Phenekos Constantinos –** ATHENS ; **Plageras Apostolos –** ATHENS ; **Politis Vasilios –** ATHENS; **Pourou Εleni –** THESSALONIKI; **Roumeliotis Christos –** ATHENS; **Routsakos Damianos –** ATHENS; **Sailer Nikolaos –** THESSALONIKI; **Sambanidou-Nikolaou Εfmorfili –** KSANTHI; **Satsoglou Aimilios –** GOUMENISSA; **Schinochoritis Panagiotis –** ATHENS; **Simelidis Dimitrios –** KOZANI; **Skoutas Dimitrios –** THESSALONIKI; **Solomonidis Ilias –** ATHENS; **Souris Sotirios –** CRETE; **Souyioultzoglou Filitsa –** THESSALONIKI; **Spanakis Emmanouil –** CHANIA; **Spina Giovanna –** ATHENS; **Spiropoulou Antigoni –** KAVALA; **Stamatakopoulos Andreas –** VOLOS; **Styliaras Ioannis –** NAFPAKTOS; **Thanasoulas Constantinos –** ATHENS; **Themeli Eleni. –** ATHENS; **Theodorou Constantina –** ATHENS; **Thomopoulos Andreas –** ATHENS; **Timosidis Michael –** KAVALA; **Troulakis Grigorios –** ATHENS; **Tsaniklidis Dimitrios –** ATHENS; **Tsatsoulis Agathoklis –** IOANNINA; **Tsimbos Stavros –** KSANTHI; **Tsiolakis Dimitrios –** THESSALONIKI; **Tsoumbeli Athanasia –** PATRA; **Tzounas Konstantinos –** THESSALONIKI; **Vamvaka Polikseni –** ATHENS; **Vlachogiannis Anestis –** DRAMA; **Vlachogiannis Nikolaos –** LAVRIO; **Vlachos Dionysios –** ATHENS; **Vourloumi Aikaterini –** AMALIADA; **Zervakis Dimitrios –** ATHENS; **Zervas Athanasios –** KALAMATA; **Τsoureka Anna –** ATHENS.
